# Decreased frequency and activated phenotype of blood CD27 IgD IgM B lymphocytes is a permanent abnormality in systemic lupus erythematosus patients

**DOI:** 10.1186/ar3042

**Published:** 2010-06-02

**Authors:** Beatriz Rodríguez-Bayona, Ana Ramos-Amaya, José J Pérez-Venegas, Carmen Rodríguez, José A Brieva

**Affiliations:** 1 Unidad de Investigación, Hospital Universitario Puerta del Mar, Avenida Ana de Viya 21, 11009 Cádiz, Spain; 2 Sección de Reumatología, Hospital de Jerez, Carretera de circunvalación s/n, 11407 Jerez de la Frontera, Spain; 3 Servicio de Inmunología, Hospital Universitario Puerta del Mar, Avenida Ana de Viya 21, 11009 Cádiz, Spain

## Abstract

**Introduction:**

Systemic lupus erythematosus (SLE) is characterized by B cell hyper-activation and auto-reactivity resulting in pathogenic auto-antibody generation. The phenotypic analysis of blood B cell subsets can be used to understand these alterations.

**Methods:**

The combined detection of CD19, CD27 and IgD (or IgM) by flow cytometry (FC) analysis delineates five well-defined blood B cell-subsets: naive, switched (S) memory, double negative (DN) memory and CD27 IgD IgM (non-switched memory) B lymphocytes, and plasma cells (PCs). This phenotypic study was performed in 69 consecutive SLE patients and 31 healthy controls.

**Results:**

SLE patients exhibited several abnormalities in the distribution of these B cell subsets, including elevated levels of DN memory B cells and PCs, and decreased CD27 IgD IgM B cells. Active SLE patients also showed decreased presence of S memory B cells and increased proportions of naive B lymphocytes. Nevertheless, when the patients in remission who did not require treatment were studied separately, the only remaining abnormality was a reduction of the CD27 IgD IgM B cell-subset detectable in most of these patients. The level of reduction of CD27 IgD IgM B cells was associated with elevated values of serum SLE auto-antibodies. Further analysis of this latter B cell-subset specifically showed increased expression of CD80, CD86, CD95, 9G4 idiotype and functional CXCR3 and CXCR4.

**Conclusions:**

The presence of a reduced blood CD27 IgD IgM B cell-subset, exhibiting an activated state and enriched for auto-reactivity, is a consistent B cell abnormality in SLE. These findings suggest that CD27 IgD IgM B lymphocytes play a role in the pathogenesis of this disease.

## Introduction

Systemic lupus erythematosus (SLE) is an autoimmune disorder with heterogeneous clinical manifestations characterized by B lymphocyte hyper-reactivity and formation of pathogenic auto-antibodies (Ab). The nature of the immune alterations causing this disease remains elusive. The occurrence of marked B cell activation and auto-reactivity leads to consideration that one, or more, of the physiological checkpoints controlling the self-antigen recognizing B cell receptor (BCR) repertoire, and the triggering and maturation of B lymphocytes, might be affected [1]. In an attempt to gain a deeper knowledge on the patho-physiology of human SLE, blood B lymphocyte subsets have been extensively explored. In this regard, when blood B cells are stained with labeled-monoclonal Ab (mAb) anti-CD19, -CD27 and -IgD (or -IgM), and analyzed by three-color flow cytometry (FC), five well-defined B cell-subsets can be identified [2]; these are: 1) naive B cells (CD19+ CD27- IgD+); 2) non-switched memory B cells, also termed CD27 IgD IgM B cells (CD19+ CD27+ IgD+); 3) conventional or switched (S) memory B cells (CD19+ CD27+ IgD-); 4) a small proportion of double negative (DN) memory B cells (CD19+ CD27- IgD-); and 5) plasma cells (CD19+ CD27++ IgD-). From a comparison of these circulating B cell-subsets between healthy controls and SLE patients, the existence of several B cell alterations in this disease has been determined. These alterations include B cell lymphopenia, increased proportion of plasma cells (PC) and DN memory B cells, decreased naive B lymphocytes and, more recently, decreased CD27 IgD IgM B cells [3-7]. The present study reveals that a marked decrease of this latter B cell-subset is a consistent and permanent blood B cell aberration in SLE patients. Further results indicate that this circulating cell-subset is enriched for cells exhibiting auto-reactivity and activation features.

## Materials and methods

### Patients and control populations

Heparinized blood samples (10 ml) were obtained from 31 normal healthy donors (16 women and 15 men; mean age 40 ± 2 years (range 26 to 59)) and 69 consecutive SLE patients (63 women and 6 men; mean age 43 ± 2 years (range 19 to 77)), who fulfilled the American College of Rheumatology criteria for SLE. Disease activity was defined by the SLE Disease Activity Index (SLEDAI). Treatments received by the patients and SLEDAI score at the time of analysis are shown in Table 1.

**Table 1:** Treatments received by the patients and SLEDAI score at the time of analysis

	Patient characteristics				
Patient	Sex	Daily prednisone dose*	Other treatment	SLEDAI**	%CD27+IgD+
1	F	LOW	HCQ	4	7
2	F	MEDIUM	CYC	16	9
3	F	HIGH	-	8	1
4	F	LOW	-	4	3
5	F	-	HCQ	2	7
6	F	LOW	-	15	3
7	F	-	HCQ	0	4
8	F	HIGH	AZA	10	5
9	F	-	-	0	8
10	F	-	HCQ	0	11
11	F	-	HCQ	0	17
12	F	LOW	MMF	10	4
13	F	HIGH	AZA	1	3
14	F	-	HCQ	2	3
15	F	MEDIUM	HCQ	8	8
16	F	HIGH	HCQ	0	13
17	F	-	-	0	8
18	M	LOW	HCQ	4	12
19	F	LOW	HCQ	2	6
20	F	LOW	-	0	2
21	F	MEDIUM	HCQ	5	7
22	F	HIGH	HCQ	19	9
23	F	LOW	-	8	2
24	F	LOW	-	4	21
25	F	-	-	0	7
26	F	LOW	AZA	1	7
27	M	LOW	AZA	0	13
28	F	-	HCQ	0	18
29	F	-	-	11	6
30	F	-	HCQ	13	3
31	F	LOW	AZA	0	4
32	M	-	-	0	6
33	F	-	HCQ	4	8
34	F	LOW	HCQ	8	4
35	M	HIGH	-	8	6
36	F	-	HCQ	0	11
37	F	-	HCQ	0	25
38	F	-	-	3	8
39	F	-	HCQ	1	27
40	F	-	AZA/RX	0	2
41	F	-	-	0	24
42	F	-	HCQ	0	4
43	F	-	-	0	6
44	F	-	MTX	0	8
45	F	-	-	0	13
46	F	-	-	0	28
47	F	-	HCQ	0	2
48	F	LOW	HCQ	0	16
49	F	-	HCQ	1	5
50	F	-	-	0	3
51	F	-	HCQ/MTX	0	8
52	F	-	HCQ	0	17
53	F	-	-	0	33
54	F	-	HCQ	0	6
55	F	LOW	MMF	4	15
56	F	-	HCQ	0	8
57	M	-	LFN	4	31
58	F	-	LFN	0	10
59	F	LOW	MTX	4	5
60	F	LOW	MTX	0	5
61	F	-	-	0	4
62	F	-	HCQ	1	7
63	F	LOW	MMF	0	15
64	F	-	-	0	3
65	F	MEDIUM	HCQ/MTX	2	2
66	M	LOW	-	0	13
67	F	LOW	-	0	13
68	F	-	-	0	17
69	F	-	AZA	1	4

*LOW (≤10 mg/day); MEDIUM (≤30 mg/day); HIGH (>30 mg/day).

AZA: azathioprine; HQC: hydroxychloroquine; CYC: cyclophosphamide; LFN: leflunomide MMF: mycophenolate mofetil; MTX: methotrexate; RX: rituximab; **SLEDAI: Systemic Lupus Erythematosus Disease Activity Index.

Patients 9, 17, 25, 29, 32, 38, 41, 43, 45, 46, 50, 53, 61, 64 and 68 had not received any treatment during a period of, at least, three months before the analysis. The sample from patient 29 was obtained just before she received cyclophosphamide bolus therapy and, consequently, her results are not included in the untreated group. Patients and healthy donors were informed of the objective of the study and gave their consent according to the Declaration of Helsinki. Approval for this study was obtained from the Institutional Review Board. (Comité Ético, Hospital Universitario Puerta del Mar).

### Preparation of peripheral blood mononuclear cells (PBMC) and FC analysis

PBMC were obtained from freshly-drawn blood by Ficoll-Hypaque (Amersham Pharmacia Biotech, Uppsala, Sweden) density gradient centrifugation. Four-colour FC analysis was performed by staining the cells with the following mAbs: peridinin chlorophyll protein-Cy5.5 (PerCP-Cy5.5)-labelled anti-CD19 (clone SJ25C1); allophycocyanin (APC)-labelled anti-CD27; phycoerythrin (PE)-labelled anti-CD95, -IgM, -CXCR3, -CXCR4, -CD49 d, -CD54, -CD62L, -CD80, -CD20, -CD22, -TACI and -CD35; fluorescein isothiocyanate (FITC)-anti-rat IgG1/2a (clone G28-5) and appropriate labelled-antibodies used as negative controls (Becton Dickinson; San Jose, CA, USA); PE and FITC-labelled anti-IgD (Dako, Hamburg, Germany), and PE-labelled anti-CD86, -CD50, -CD21 (Beckman Coulter; Fullerton, CA, USA). 9G4 rat mAb was a generous gift by Professor F Stevenson (Southampton University Hospitals Trust, UK). Incubation of the cells (10^6 ^PBMC/100 μl of phosphate buffer saline (PBS)) with mAbs was performed at room temperature for 15 minutes. FC analysis was performed with a FACSCalibur cytometer using CellQuest software (Becton Dickinson; San Jose, CA, USA). At least 20,000 CD19+ cells were collected for each analysis.

### Chemotaxis assays

Chemotaxis to several chemokines of CD27 IgD IgM B cells from control and SLE individuals was determined as previously described [8]. Briefly, chemotaxis assays were carried out in 24-well plates with transwell inserts (5-μm pore size; Costar Corning, Corning, NY, USA). RPMI 1640 medium supplemented with BSA 0.5% was used as assay medium. PBMCs were diluted in medium at a concentration of 10 × 10^6 ^cells/mL. The lower transwell chamber was filled with 600 μL of medium either alone (control) or containing 1 μg/mL of CXCL12 (SDF-1α), CXCL10 (IP-10) or CCL3 (MIP-1α) (PeproTech, London, UK), and then 100 μL of the cell suspension was added to the upper chamber. Cells were allowed to migrate for four hours at 37°C. Finally, the cells were collected from the lower chamber and were stained and quantified by FACS. The total number of migrated CD19+ CD27+ IgD+ B cells was evaluated. Specific migration was calculated as the number of cells that migrated in response to the stimuli divided by the number of cells that migrated in response to medium (migration index).

### Autoantibody detection

Anti-nuclear antibodies (ANA) were determined in patients' serum samples obtained at the moment of the study using indirect immunofluorescence (IIF) on HEp-2 cells. Anti-dsDNA Ab were measured by ELISA (*AESKULISA *dsDNA G, AESKU DIAGNOSTICS, Wendelsheim, Germany). Several nuclear specificities including U1-RNP, Sm, Ro/SSA (52 and 60 kDa proteins), La/SSB, Scl-70 topoisomerase-I, Ribosomal P-proteins and CENP-B were detected by immunodot (INNO-LIA ana, INNOGENETICS, Ghent, Belgium).

### Statistical methods

**Figure 1:**
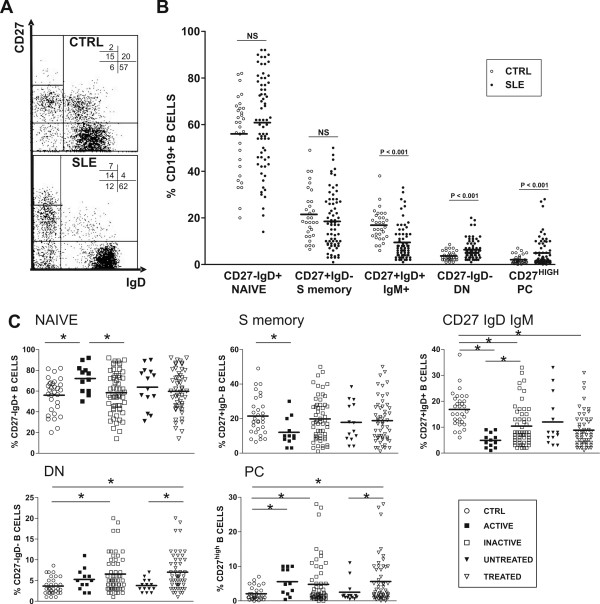
**Comparative analysis of blood B cell-subsets from SLE patients and healthy controls**. CD19+ cells can be distributed into different B cell-subsets according to their additional expression of CD27 and IgD. **A**. Representative CD27-IgD dot plots from a control and a SLE patient indicating the distribution and percentages of these B cell-subsets: CD27-IgD+ (naïve), CD27+IgD- (S memory), CD27+IgD+ (CD27 IgD IgM), and CD27-IgD- (DN memory) B lymphocytes, and CD27^high^IgD^- ^cells (PC). **B**. Scatter plots represent the percentages of these B cell-subsets in 31 controls (open circles) and 69 SLE patients (closed circles). The mean of each set of values is shown as a horizontal line. *P *values (Student's *t *test) are included. **C**. Scatter plots represent the percentage of each B cell-subset in controls and in SLE patients distributed according to disease activity (12 active and 57 inactive), and to the treatment (54 treated and 14 untreated). Symbols representing the control and the different SLE subgroups are displayed (bottom right panel). *P *values were calculated for the difference between the two pairs of SLE subgroups and between SLE subgroups and the control group. *P *values lower than 0.05 were considered statistically different (indicated as *).

Differences between groups were analyzed with the Student's *t *test and Mann-Whitney test. Comparison of data included in Figure 1C was performed by ANOVA testing, followed by Tukey's post-hoc analysis for pairwise comparisons. *P-*values lower than 0.05 were considered statistically significant.

## Results

### Blood B cell-subsets alterations in SLE patients

Blood B cells, including PCs, can be identified as CD19+ cells [2]. The absolute number of blood B cells (CD19+ cells) in the present group of SLE patients was significantly lower than in normal subjects (82.0 ± 8.2 cells/μl versus 144.7 ± 29 cells/μl, for SLE and healthy controls, respectively; mean ± SEM; *P *< 0.01). The additional staining of these cells for surface CD27 and IgD molecules, and the subsequent FC analysis, allows the distinction of five B cell-subsets. Figure 1A shows an example of the phenotypic analysis of these blood B cell-subsets in a control individual and in one SLE patient (FC dot plots), and Figure 1B summarizes the results obtained from all the SLE patients and the healthy controls. As can be seen, SLE samples showed several alterations in the distribution of these B cell-subsets, including increased proportions of DN memory B cells (CD27- IgD-) and PC (CD27++ IgD-) and decreased CD27 IgD IgM B cells (CD27+ IgD+); this latter finding was the most consistent. The results for blood naive B cells (CD27- IgD+) and S memory B cells (CD27+ IgD-) were similar in SLE patients and controls.

### Effect of disease activity and remission

The effect of disease activity on the described alterations of the B cell-subsets was examined next. Accordingly, SLE patients were distributed into two groups: one group consisted of inactive and mildly active cases (SLEDAI range 0 to 5; 0.95 ± 0.2, mean ± SEM; N = 57); the second group consisted of moderate to highly active patients (SLEDAI range 8 to 19; 11.17 ± 1.09, mean ± SEM; N = 12). Figure 1C shows that both groups exhibited abnormally high figures of DN memory B cells and PC, but they differed in that the more active SLE cases additionally showed significantly lower CD27 IgD IgM B cells and S memory B cells, and abnormally high naive B cells.

The relevance of the described B cell-subset imbalances during the course of the disease was then analyzed. To this end, the group of SLE patients that remained in a controlled and favorable phase of the disease, requiring no treatment at the time of the study, was explored separately, and compared with the group of patients receiving treatment. The clinical characteristics of the group of untreated patients are depicted in Table 2.

**Table 2:** Summary of clinical characteristics of the group of untreated patients

Patient number*	P9	P17	P25	P32	P38	P41	P43	P45	P46	P50	P53	P61	P64	P68
Sex	F	F	F	M	F	F	F	F	F	F	F	F	F	F
**CD27+IgD+ (%)**	8	8	7	6	8	24	6	13	28	3	33	4	3	17
SLEDAI** score	0	0	0	0	3	0	0	0	0	0	0	0	0	0
SLICC*** score	0	0	1	0	1	2	1	0	0	5	0	0	1	0
Disease manifestations****:														
**Joint**	x	x	x	x		x	x	x	x		x	x	x	x
**Mucocutaneous**	x	x		x		x		x	x	x	x			x
**CNS involvement**			x			x								
**Renal involvement**		x				x				x				
**Hematologic**	x													
**Serositis**					x			x						

(*) Patients' numbers are the same as in Table 1. (**) SLEDAI, Systemic Lupus Erythematosus Disease Activity Index. (***) SLICC, "Systemic Lupus International Collaborating Clinics" damage index for SLE. Except where indicated otherwise, data were obtained at the moment of the inclusion into the study. (****) The clinical manifestations that appeared throughout the course of the disease are shown.

CNS: central nervous system

Figure 1C shows that, the elevation of DN memory B cells and PC proportions previously observed when all the SLE cases were considered (Figure 1B), was normalized in the group of untreated patients. In contrast, most of the patients included in the untreated group (9 out of 14) still showed a decrease in the proportion of CD27 IgD IgM B lymphocytes (Figure 1C), although, as a whole, the difference between this group and the controls was non-significant (Tukey's test, *P *= 0.09).

### Correlation between blood CD27 IgD IgM B cells and serum SLE auto-Ab level

**Figure 2:**
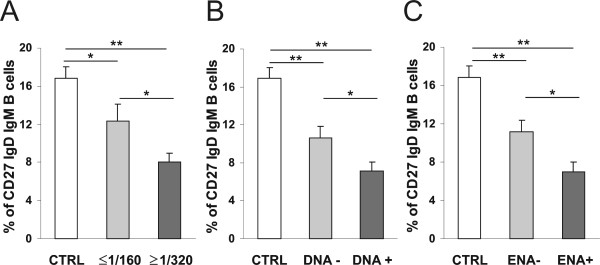
**Comparative analysis of blood CD27 IgD IgM B cell-subsets from healthy controls and different groups of SLE patients according to the presence of autoantibodies**. **A**. B cell subsets from healthy controls (n = 31) and SLE patients with negative or low levels (IIF titre ≤1/160; n = 23) and medium or high levels (IFF titre ≥1/320; n = 46) of ANA were compared. **B **and **C**. Comparison among healthy controls and SLE patients with anti-dsDNA negative (n = 46) or positive (n = 23) and anti-ENA negative (n = 41) or positive (n = 28) Ab, respectively. Differences between groups are indicated (* *P *< 0.05; ** *P *< 0.01).

The relationship between the reduction of CD27 IgD IgM B lymphocytes and the level of serum SLE auto-Ab was also investigated in these patients. Accordingly, percentages of CD27 IgD IgM B cells in SLE patients with negative/low levels (IIF titre ≤1/160) and medium/high levels of ANA (IIF titre ≥1/320) (Figure 2A), positive and negative anti-dsDNA Ab (Figure 2B), and positive and negative anti-ENA (extractable nuclear antigens) Ab (Figure 2C), were determined. Figure 2 shows that higher levels of ANA, anti-dsDNA and anti-ENA Ab were associated with lower numbers of the B cell subset under study (Figure 2A, B and 2C, respectively).

### Phenotypic and functional characteristics of SLE CD27 IgD IgM B lymphocytes

**Figure 3:**
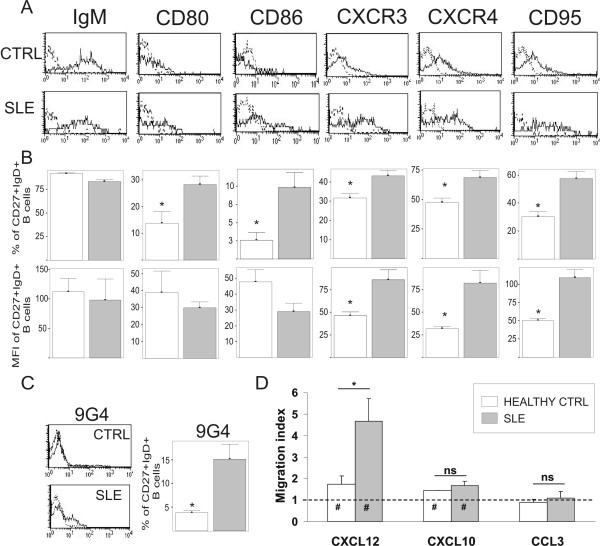
**Comparative phenotypic analysis of blood CD27 IgD IgM B cell-subsets (CD27+IgD+) from controls and SLE patients**. This B cell-subset was examined for the surface expression of different molecules. **A**. Representative histograms depict the expression of IgM, CD80, CD86, CXCR3, CXCR4 and CD95 for a healthy control and a SLE patient. For each baseline plot, negative isotypic antibody controls are superimposed in dotted lines. **B**. Bar histograms show the percentages of positive CD27+IgD+ B cells (upper) and the mean fluorescence intensity (lower) for each marker in healthy controls (N = 10; open bars) and SLE patients (N = 10; grey bars). **C**. Expression of 9G4 idiotype in healthy controls (N = 6) and SLE patients (N = 22) is shown. A representative histogram is shown. Results represent the mean ± SEM. *P *values were calculated using the Mann-Whitney test. *P *values lower than 0.05 were considered statistically different (marked with an asterisk). **D**. A comparison is shown of the chemotaxis of CD27 IgD IgM B cells from healthy controls and SLE patients induced by CXCL12, CXCL10 and CCL3. Migration in the absence of stimuli is represented as a dotted line. Results are expressed as a migration index and represent the mean ± SEM (N = 4). *P-*values were calculated using the Mann-Whitney test. *P *< 0.05 was considered statistically significant. Asterisks represent significant differences between healthy controls and SLE patients. Hashes indicate significant differences in chemokine-induced migration with respect to medium alone.

**Figure 4:**
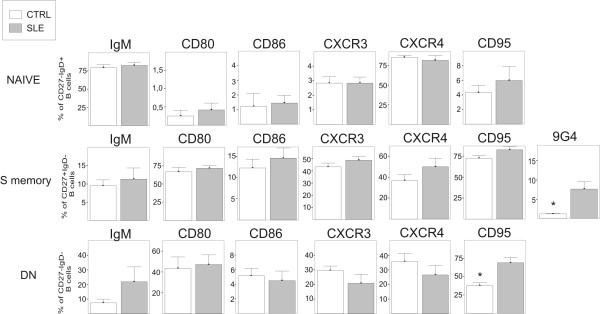
**Comparative phenotypic analysis of different blood B cell-subsets from controls and SLE patients**. Indicated B cell-subsets were examined for the surface expression of several molecules including CD80, CD86, CXCR3, CXCR4, CD95 and 9G4. Bar histograms show the percentages of positive B cells (mean ± SEM) for each marker in healthy controls (N = 10; open bars) and SLE patients (N = 10; grey bars). *P *values were calculated using the Mann-Whitney U test, and statistically significant p values are marked with an asterisk (*P *< 0.05).

In an attempt to gain a deeper insight into this particular B cell-subset, a broad phenotypic analysis was performed on them, and the results obtained in SLE patients and healthy controls were compared. The phenotype of normal CD27 IgD IgM B lymphocytes has been in part previously reported [2,9,10]. Figure 3A shows an example of the histogram of expression of several B cell markers on CD27 IgD IgM B cells, and Figure 3B summarizes the results obtained in all the SLE and control blood samples analyzed. The majority of SLE and control CD27 IgD IgM B cells similarly expressed CD19, CD20, CD21, CD22, CD35, CD49 d, CD50, CD54, CD62L and TACI (data not shown) and surface IgM. In contrast, Figure 3A and 3B show that SLE CD27 IgD IgM B cells expressed higher levels of the co-stimulatory molecules CD80 and CD86, the death receptor CD95, and the chemokine receptors CXCR3 and CXCR4. Figure 3C also shows that the frequency of 9G4+ cells detected in this B cell-subset was clearly higher in SLE than in controls. This distinctive pattern of molecule expression was equally detected in CD27 IgD IgM B cells from patients with active disease or in remission stage (data not shown). Interestingly, the comparison of the same phenotypic study performed on naive lymphocytes and DN and S memory B cells from healthy controls and SLE patients revealed that, they were appreciably similar, with the exception of a higher proportion of CD95-expressing DN memory B cells in SLE patients (Figure 4). Therefore, the observation of relevant differences in phenotype between SLE and control blood B cells was essentially restricted to the CD27 IgD IgM B cell-subset. Finally, the functionality of increased CXCR4 and CXCR3 expression on CD27 IgD IgM B cells in SLE patients was examined in a chemotaxis assay. As can be seen in Figure 3D, CD27 IgD IgM B cells from healthy controls and SLE patients migrated to the CXCR4 ligand CXCL12, although these latter cells exhibited markedly higher activity (*P *< 0.02). In addition, cells of healthy control and SLE patients also migrated to CXCL10, a ligand of CXCR3. Again, the chemotactic activity of SLE CD27 IgD IgM B lymphocytes was higher, although, in this case, the observed increase was not significant. SLE and healthy control CD27 IgD IgM B cells did not express the chemokine receptor CCR5 (data not shown) and, as expected, no chemotaxis was observed when CCL3, a ligand of CCR5, was tested.

## Discussion

The present study demonstrates that the distribution of blood B cell-subsets in SLE patients exhibits a variety of alterations including elevated proportions of DN memory B lymphocytes and PC, and decreased CD27 IgD IgM B lymphocytes. In addition, active patients show decreased S memory and increased naïve B lymphocytes, indicating that patients in more active phases exhibit more abnormalities in the distribution of these B cell-subsets, and these abnormalities are more pronounced. These data are consistent with previous reports [3,5-7] and, taken together, indicate that, in SLE patients, the B cell compartment undergoes profound alterations and imbalances that are noticeable in the distribution of the normal B cell-subsets present in the circulation. Moreover, the results observed in the group of patients in remission (untreated patients, Table 2) show that a decreased proportion of CD27 IgD IgM B lymphocytes is the only B cell-subset alteration persisting in the majority of these cases. Serum SLE auto-Ab are relevant clinical parameters in this disease. Present data reveal that the level of elevation of these auto-Ab correlates with the intensity of reduction of CD27 IgD IgM B lymphocytes in this disease. Further analysis indicates that CD27 IgD IgM B cells from SLE patients exhibit higher expression of CD95, CD80, CD86, CXCR3 and CXCR4. Chemotaxis assays confirm that the increased expression of CXCR4 and CXCR3 observed in SLE CD27 IgD IgM B cells is functional, as the migration capacity to the appropriate ligands exhibited by these lymphocytes is higher in SLE patients' than in healthy controls. Increased CD95 expression has been previously reported in blood DN memory B cells from SLE patients [11]. Present results show that this observation can be also extended to CD27 IgD IgM B cell-subset (Figures 1 and 4). In addition, increased expression of CD80 and CD86 in certain SLE circulating B lymphocytes has been previously reported [12], although the use of a B cell marker selection profile different from that employed in the present study makes the comparison of these results difficult. Present data are consistent with the notion that the SLE patients' CD27 IgD IgM B cells distinctively are in an activated state, exhibiting a potentially higher capacity to migrate and to interact with T cells. Taken together, the present findings indicate that a decreased proportion of blood CD27 IgD IgM B lymphocytes appears to be an alteration that is permanent in SLE patients, irrespective of disease activity; in addition, these cells exhibit an activated phenotype. These observations suggest that CD27 IgD IgM B cell-subset might play a role in the patho-physiology of SLE.

Previous studies have established that the repertoire of mature naïve B lymphocytes is enriched for self-reactive clones in SLE patients [13,14]. This observation indicates that SLE patients exhibit defective B cell tolerance checkpoints present in normal subjects [15] and, in consequence, the emergence of an increased frequency of these auto-reactive naive B cells is probably relevant in the pathogenesis of SLE. However, a possible role of CD27 IgD IgM B lymphocytes in the state of auto-reactivity characteristic of this disease has been less studied. This is, at least in part, due to the fact that the origin, nature and functional significance of normal CD27 IgD IgM B cells remain subject of debate [7,9,10]. These lymphocytes were defined as non-switched (IgM+ IgD+) memory B cells based on the findings that they express the putatively memory B cell marker CD27 [2], and harbor *IGV *genes exhibiting a low but detectable number of somatic mutations, an event classically restricted to post-germinal center (GC) memory B cells [2,9]. Nevertheless, the observation of normal quantities of mutated CD27 IgD IgM B lymphocytes in immunodeficient patients that lack GC formation and conventional switched memory B cells, indicates that the generation of the cell-subset under study can be GC-independent; hence, the *IGV *gene somatic mutations present in this B cell-subset are thought to represent a pre-immune diversification process [9,16]. Human CD27 IgD IgM B lymphocytes have been associated with the spleen marginal zone, and they appear to be involved in the production of natural antibodies [9,16]. The mechanisms that determine the somatic mutations occurring in CD27 IgD IgM B cells remain to be clarified, although it has been recently shown that this B cell subset can express activation-induced cytidine deaminase (AID), either during a transient post-natal phase [17], or upon Toll Like Receptors (TLR)-engagement [18]. Under this latter condition, CD27 IgD IgM B cells are capable of differentiating into IgG-secreting plasma cells [18]. Interestingly, normal human CD27 IgD IgM B lymphocytes exhibit a frequency of auto-reactive BCR much lower than that observed in naive B lymphocytes, suggesting that an additional checkpoint for preventing self-reactivity exists at this level [19]. In this context, it is reasonable to think that a failure of this BCR control process would necessarily lead to the emergence within this B cell-subset of auto-reactive clones. It is well-established that the immunoglobulin (Ig) idiotype recognized by the 9G4 mAb is enriched in auto-reactive B cell populations, including anti-DNA Ab-bearing B cells occurring in SLE patients [20]. Accordingly, the presence of B lymphocytes containing the 9G4 idiotype in their surface Ig was examined in the blood CD27 IgD IgM cell-subset. Present data reveal that the frequency of 9G4+ cells detected in this B cell-subset was, on average, five times higher in SLE than in controls. This result indicates that SLE patients show a defective control on the appearance of auto-reactive clones within the circulating CD27 IgD IgM B cell-subset. The cause of this failure remains unknown. It is conceivable that these auto-reactive cells, after appropriate self-antigen recognition, maybe in combination with TLR-activation [18], would undergo activation and migration toward lymphoid tissue where they could progress into further differentiation, giving rise to auto-reactive switched memory B cells and PCs. This explanation is consistent with the findings reported here. Thus, a permanent decrease of circulating CD27 IgD IgM B cells could be the result of self-antigen activation of auto-reactive B cells contained in elevated proportions in this subset, and their subsequent recruitment into lymphoid tissues. In fact, 9G4+ activated B cells and PCs have previously been detected in lymphoid tissues (spleen, tonsil GC and bone marrow) from SLE patients, but not from normal subjects [21]. In conclusion, CD27 IgD IgM B lymphocytes might play a role in the complex B cell alteration causing SLE.

## Conclusions

This study shows that the presence of a reduced blood CD27 IgD IgM B cell-subset, exhibiting an activated state, an increased capability to migrate towards CXCR4 ligand and enriched for auto-reactivity is a prominent B cell abnormality in SLE. In addition, higher levels of ANA, anti-dsDNA and anti-ENA Ab were associated with lower numbers of CD27 IgD IgM B cells in SLE patients. These findings suggest that CD27 IgD IgM B lymphocytes play a role in the pathogenesis of this disease.

## Abbreviations

AID: activation-induced cytidine deaminase; APC: allophycocyanin; Ab: antibody; ANA: anti-nuclear antibodies; BCR: B cell receptor; DN: double negative; ENA: extractable nuclear antigens; FC: flow cytometry; FITC: fluorescein isothiocyanate; GC: germinal center; IIF: indirect immunofluorescence; PerCP-Cy5.5: peridinin chlorophyll protein-Cy5.5; PBMC: peripheral blood mononuclear cells; PCs: plasma cells; PE: phycoerythrin; S: memory; SLE: systemic lupus erythematosus; SLEDAI: SLE disease activity index; TLR: toll like receptors.

## Competing interests

The authors declare that they have no competing interests.

## Authors' contributions

BRB carried out the acquisition of data, analysis and interpretation of data and statistical analysis. ARA participated in the acquisition of data. JJPV participated in the acquisition of data and in patients' collection. CR participated in analysis and interpretation of data and manuscript preparation. JAB performed the study design and the manuscript preparation. All authors read and approved the final manuscript.
